# Dracohodin Perochlorate Stimulates Fibroblast Proliferation via EGFR Activation and Downstream ERK/CREB and PI3K/Akt/mTOR Pathways In Vitro

**DOI:** 10.1155/2019/6027186

**Published:** 2019-08-25

**Authors:** Lin Liu, Xiaowen Jiang, Wenhui Yu

**Affiliations:** ^1^College of Veterinary Medicine, Northeast Agricultural University, Harbin 150030, China; ^2^Key Laboratory of the Provincial Education Department of Heilongjiang for Common Animal Disease Prevention and Treatment, Harbin, China

## Abstract

In recent years, an increasing number of natural plant extracts have been determined to be potential drugs for various illnesses. In this study, we investigated the effects of dracorhodin perchlorate (DP) on fibroblast proliferation, which is crucial for wound healing. Cell proliferation assays were performed by different concentrations of DP, and the cell viability was detected by CCK-8 kits. After DP treatment for 24 h, the cell cycle was checked by flow cytometer. EGFR and downstream signaling pathways ERK1/2 and PI3K were examined with DP treatment by western blot. We further determined the effects of the related inhibitors on DP-induced relative protein phosphorylation and cell proliferation. The results showed that 3 *μ*g/mL of DP promoted cell proliferation most significantly at treatment lengths of 24 h, and the percentage of cells in the S + G2 phase increased compared to those of the control group. In western blot detection, we found that DP significantly upregulated EGFR phosphorylation and activated the downstream ERK/CREB and PI3K/Akt/mTOR signaling pathway. Moreover, the results also showed that AG1478 abolished DP-induced relative protein activation and cell proliferation. When U0126 or LY294002 pretreated cells alone, DP-induced p-ERK or p-PI3K downstream proteins and cell proliferation were suppressed compared to those of the control group, but EGFR was not affected. In addition, ICG001 and BEZ235 collectively eliminated DP-induced fibroblast proliferation. Our findings suggest that DP-promoted fibroblast proliferation is stimulated by p-EGFR-induced activation of the ERK1/2-CREB and PI3K/Akt/mTOR pathways. Our present study explored the mechanism of DP-promoted fibroblast proliferation and provided a new basis for wound healing.

## 1. Introduction

Skin damage is common in clinical surgery. Further, wound healing is a complex biological process. Skin damage initiates a series of biological reactions toward self-healing, including hemostasis, granulation tissue regeneration, and tissue reconstruction [1]. However, a larger area of cutaneous deficiency requires more time to heal and increases the risk of wound infection. Therefore, great value is placed on researching methods to speed up wound healing. When damage occurs to the skin, various cells and cellular functions are working together in the healing process. In the early stages of wound healing, neutrophils can reduce the inflammation caused by injury [2]. Endothelial cells contribute to blood vessel formation [3]. Fibroblasts are the most vital cells in the wound healing process because they synthesize and secrete a large number of collagen fibers and matrix components, which combine with the capillaries to form granulation tissue. It is beneficial for the epidermal cells to cover the wound [4]. In addition, some studies have shown that fibroblasts are also important for fracture healing. Fibroblasts not only proliferate strongly, but can also synthesize and secrete collagen I, which forms a huge fibrous callus link to fracture ends [5]. One report stated that skin fibroblast-mediated bone morphogenetic protein-2 (BMP2) therapy may be considered as a potential treatment for various types of fractures and bone defects [6].

**Figure 1:**
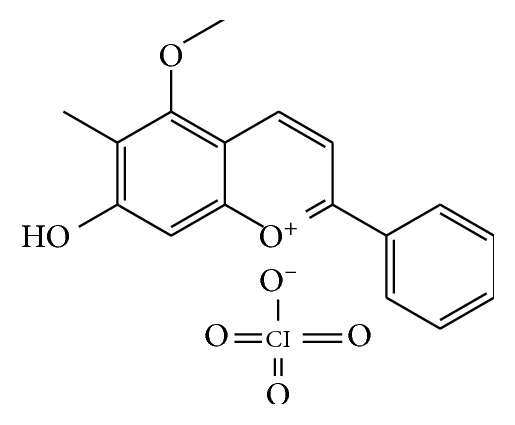
Structure of DP.

With the rapid development of medical field research, an increasing number of plants are being studied in the treatment of diseases. The most well known of them is artemisinin, extracted from the stem of *Artemisia annua*, which has saved the lives of millions of malaria patients [7]. In addition, ginseng extract is widely used for cancer treatment [8], berberine can inhibit bacterial growth [9], and morphine has analgesic effects [10]. Dragon's blood is a resin obtained from the fruit of a number of palmae plants predominantly distributed throughout Asia and Indonesia [11]. Dragon's blood is used for the improvement of blood circulation and reducing pain [12]. Research also indicates that dragon's blood has a variety of pharmacological effects, including immunoregulatory [13], antidiarrheic [14], antibacterial [15], antiviral [16], antioxidant [17], and anticancer [18]. Dragon's blood extracts contain a variety of active ingredients, mainly flavonoids and resin grade. One extract of dragon's blood, dracorhodin, is usually present in salt form, named dracorhodin perchlorate (DP) (Figure 1) [19].

Recent research has demonstrated that DP promotes vascular endothelial cell proliferation and angiogenesis to accelerate wound healing [20]. However, research on whether DP can promote fibroblast proliferation has not been reported. Moreover, the mechanism of DP-induced cell proliferation remains unclear.

EGFR and FGFR are extensively embedded in the cell membrane. They are sensitive to external stimuli. The EGFR signaling pathway plays an important role in cell processes, such as cell growth, proliferation, and differentiation [21]. Dracorhodin is a flavonoid compound extracted from dragon's blood [22]. It has been reported that flavonoids activate EGFR signaling pathways [23]. Thus, we hypothesized that DP may stimulate fibroblast proliferation via EGFR. Cell proliferation caused by EGFR activation mediates two main transmission methods to the nucleus: Ras/Raf/MAPK and the PI3K/PKC/IKK signaling pathways. When the signal is transmitted to the nucleus, the transcriptional level of the gene is increased. This causes cell proliferation and transformation [24]. ERK and JNKs are members of the mitogen-activated protein kinase (MAPK) family, and their activation is crucial to the regulation of cell growth [25]. Studies have shown that CREB, a transcription factor, is involved in cell proliferation and activated by phosphorylation of ERK [26]. Similarly, PI3K protein family also plays a key role in cell proliferation, differentiation, apoptosis, and other cell function regulation [27]. In this study, we examined the effects of DP on cell proliferation and the cell cycle. Moreover, the relative signaling pathway was detected with or without the inhibitor pretreatment. The purpose of this study was to determine the mechanism of DP-induced fibroblast proliferation and provide a basis for further clinical application.

## 2. Materials and Methods

### 2.1. Cell Viability and Cell Proliferation Assays

DP was purchased from the National Institutes for Food and Drug Control (110811, Beijing, China). NIH/3T3 fibroblast lines were purchased from the Cell Bank of the Chinese Academy of Sciences (Shanghai, China). We followed the methods of Jiang et al. [28]. Fibroblasts at passage 5 to 9 were used experimentally. Trypsin (0.25%, Gibco, Germany) was used to digest fibroblasts until the cells were all resuspended. Fibroblasts were seeded into 96-well plates at a density of 1 × 10^4^ cells per well and incubated for 12 h in Dulbecco's modified Eagle's medium (DMEM) (Hyclone, USA) containing 10% fetal bovine serum (FBS) (complete medium) (Gibco, Germany) at 37°C in a humidified, 5% CO_2_ atmosphere. After 12 h of incubation, the complete medium was removed from each well and replaced with DMEM in the absence of FBS (starvation medium) prior to a further 3 h incubation. After starvation for 3 h, complete medium containing DP at concentrations of 0 (control), 0.5, 1, 1.5, 2, 2.5, 3, 3.5, and 4 *μ*g/mL (*n* = 5 wells in triplicate plates) was added into every well with 24 h treatment. In addition, 50 ng/mL epidermal growth factor (EGF) was regarded as the positive control group. The Cell Counting Kit-8 (CCK-8) assay was used to assess cell viability. Viable cells were measured at an absorbance (Abs) of 450 nm using an enzyme-linked immune detector (Gene, China). Effective concentrations of DP were identified through the above experiment. NIH/3T3 cells were also treated with 0 (control), 2, 3, and 4 *μ*g/mL of DP for 0, 6, 12, 24, and 36 h separately, and cell proliferation was determined using the CCK-8 as described previously. Cell viability was determined as follows:

(1) $$\begin{array}{c} \text{cell viability}\left(\%\right)=\frac{\text{Abs of DP treated group}}{\text{Abs of control group}}\times 100\%. \end{array}$$

### 2.2. Flow Cytometry

Fibroblasts were seeded into cell-cultured plates at a density of 1 × 10^5^ cells and incubated for 12 h in complete medium at 37°C in a humidified, 5% CO_2_ atmosphere. After 12 h of incubation, the complete medium was removed from each well and replaced with starvation medium prior to a further 3 h incubation. After starvation for 3 h, complete medium containing DP at concentrations of 0 (control), 2, 3, and 4 *μ*g/mL was added into every dish with 24 h treatment. Flow cytometry analysis was performed using a Cell Cycle and Apoptosis Analysis Kit (Beyotime, Shanghai, China) according to the manufacturer's instructions. In brief, the cells were washed with ice-cold phosphate-buffered saline (PBS) and fixed in 70% ice-ethanol for 6 h at a concentration of 1 × 10^6^ cells/ml. Then, cells were stained with propidium iodide for 30 min at 37°C in the dark. A flow cytometer (Becton Dickinson, San Jose, CA) was used to detect cell cycle changes. The cell proliferation index (proliferation index, PI = S + G2/M) represents the number of proliferating cells in the cell population. All experiments were performed in triplicate.

### 2.3. Western Blot Analysis for Related Proteins

Expressions of related proteins in NIH/3T3 fibroblasts were determined after treatment with 3 *μ*g/mL DP for 0, 5, 10, 15, 30, and 60 min. At each time point, the total proteins in cells were harvested, and the protein concentrations were detected using an Enhanced BCA Protein Assay Kit (Beyotime, Shanghai, China). The rest of the cell suspension was then aspirated and mixed with the sodium dodecyl sulfate-polyacrylamide gel electrophoresis (SDS-PAGE) loading buffer in a ratio of 1 : 4. Equal amounts of proteins were separated by SDS-PAGE and electrotransferred to polyvinylidene fluoride (PVDF) membranes. The PVDF membranes were incubated in Tris-buffered saline (TBS) containing 5% skimmed milk and 0.1% Tween-20 for 60 min and blotted with primary antibodies at 4°C overnight. The following primary antibodies (all antibodies were purchased from Absin Bioscience Co., Ltd., Shanghai, China) were used: anti-phospho-(EGFR, FGFR, ERK1/2, JNK, CREB, PI3K, Akt, and mTOR), anti-(EGFR, FGFR, ERK1/2, JNK, CREB, PI3K, Akt, and mTOR), and anti-GAPDH (glyceraldehyde-3-phosphate dehydrogenase). All antibodies were diluted (1 : 500) according to the instructions. The membranes were incubated for 2 h with anti-mouse or anti-rabbit horseradish peroxidase-conjugated secondary antibodies (1 : 2000, Absin Bioscience Co., Ltd., Shanghai, China). Reaction products were visualized by detection of chemiluminescence using the ECL Western Blotting Detection System (GE Healthcare, Piscataway, NJ, USA). Quantification of the relative band gray intensity was performed by scanning densitometry using ImageJ software (National Institutes of Health, Bethesda, MD, USA). The phosphorylation level was calculated by the following formula:

(2) $$\begin{array}{c} \text{phosphorylation level}=\frac{\text{gray intensity of Phospho protein}}{\text{gray intensity of protein}}. \end{array}$$

### 2.4. Effects of Inhibitors on DP-Induced Related Protein Phosphorylation

To further investigate the relationship among the activated proteins, specific signaling pathway inhibitors were used to pretreat cells before adding DP into the cell-cultured dish. The inhibitors described below were all purchased from Absin Bioscience Co., Ltd., Shanghai, China. According to the manufacturer's instructions, AG1478, U0126, or LY294002 was dissolved in the complete medium and maintained at a final concentration of 10 *μ*M. Cells were seeded into cell-cultured plates at a density of 1 × 10^5^ cells and cultured by complete medium containing inhibitors for 12 h. After that, the medium was replaced by complete medium containing 3 *μ*g/mL of DP. Then, the cells were cultured for 0, 5, 15, and 30 min. At each point, related proteins were harvested and detected as previously described.

### 2.5. Effects of Inhibitors on DP-Induced Cell Proliferation

To determine the involvement of EGFR, ERK1/2-CREB, and PI3K/Akt/mTOR activation in cellular proliferation by DP, we measured the DP-induced fibroblast proliferation by CCK-8 assays using the related inhibitors (AG1478, U-0126, LY294002, ICG001, and BEZ235, respectively). Initially, we detected the effects of inhibitors on cell proliferation. Fibroblasts were treated with inhibitors, respectively, for 24 h, and the cell viability was then measured by CCK-8.

In further experiments, the experimental groups were divided into eight groups: DP-treated group without inhibitor pretreatment (DP group), DP + AG1478, DP + U0126, DP + LY294002, DP + U0126 + LY294002, DP + ICG001, DP + BEZ235, and DP + ICG001 + BEZ235. The cells without DP treatment were used as the control group. Cells were pretreated with inhibitors separately for 12 h and then were cultured in a complete medium containing 3 *μ*g/mL DP for 24 h. Finally, the cell viability was assessed using CCK-8.

### 2.6. Statistical Analysis

All results are reported as mean ± SD. The *t*-test was used to identify differences between two groups, and one-way analysis of variance (ANOVA) was used to identify differences among multigroups followed by LSD test (SPSS 17.0 software). Differences with *P* < 0.05 were considered statistically significant, and differences with *P* < 0.01 were considered extremely significant.

## 3. Results

### 3.1. DP Promoted Fibroblast Proliferation

**Figure 2:**
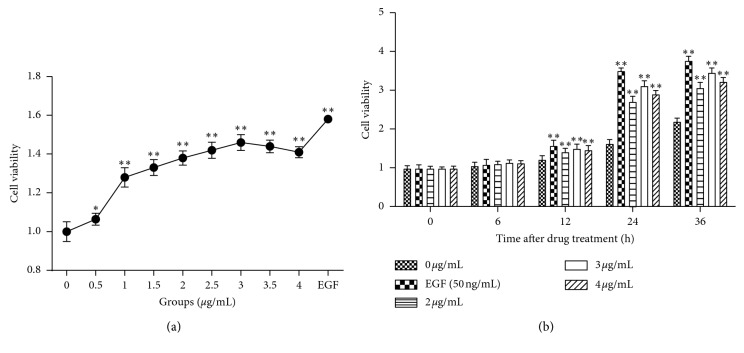
DP promotes fibroblast proliferation. Cells in 96-well plates were cultured with different concentrations of DP for 24 h. (a) Cell viability was measured by CCK-8 assay, and the results showed that DP (>0.5 *μ*g/mL) effectively promoted cell proliferation, with a peak at 3 *μ*g/mL. (b) Fibroblasts were treated by DP (0, 2, 3, and 4 *μ*g/mL) for 0, 6, 12, 24, and 36 h, respectively. We determined that DP stimulated cell proliferation in a time-dependent manner (data are presented as mean values ± SD, *n* = 15 for each bar; ^*∗*^*P* < 0.05; ^*∗∗*^, ^##^ and ^●●^*P* < 0.01 vs. control group at the same time).

Fibroblasts were treated with different concentrations (0.5, 1, 1.5, 2, 2.5, 3, 3.5, and 4 *μ*g/mL) of DP for 24 h. Cells in the control group were treated with a complete medium. Then, the cell viability was detected for each group. As shown in Figure 2(a), we found that EGF (positive control group) significantly stimulated fibroblast proliferation (*P* < 0.01 vs. control group) and DP promoted fibroblast proliferation significantly compared with that of the control group when DP was more than 0.5 *μ*g/mL (*P* < 0.01), with a peak at 3 *μ*g/mL. In addition, cell viability was weakened when the cells were treated with higher concentrations (>3 *μ*g/mL) of DP. In cell proliferation assays, we found that cell viability in the drug-treated group was significantly higher than that of the control group after 12 h of treatment (*P* < 0.01). Moreover, under DP treatment, cell proliferation was enhanced in a time-dependent manner. At 24 h, DP-induced cell proliferation was the most obvious. In these three drug-treated groups, DP at 3 *μ*g/mL was identified as the most effective concentration (Figure 2(b)), which was used for subsequent experiments.

### 3.2. DP Promoted Cell Cycle Progression in Fibroblast Cells

**Figure 3:**
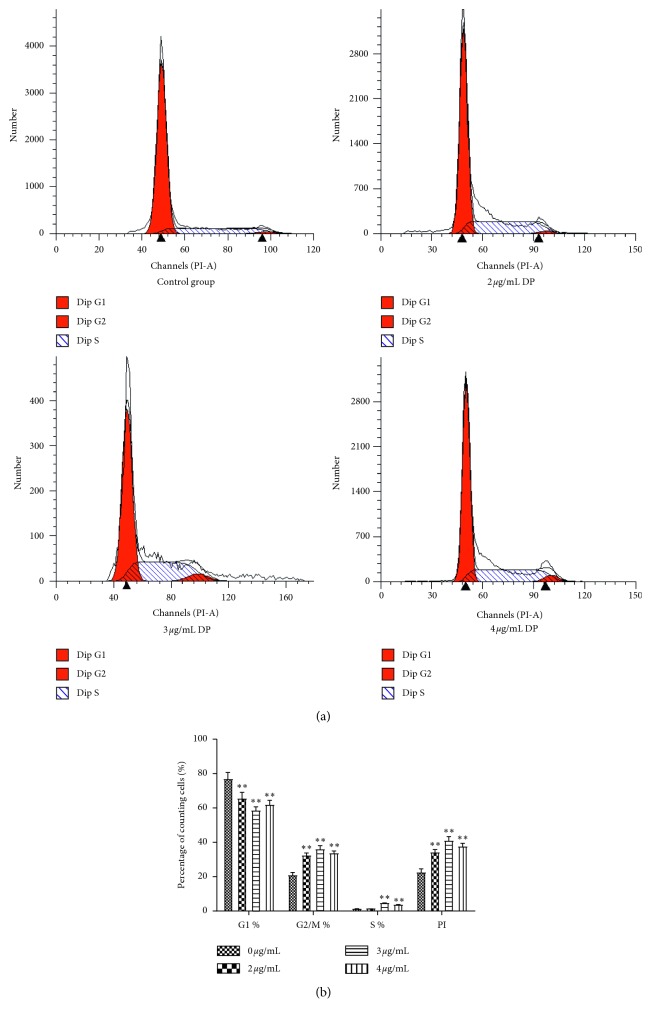
DP promotes cell cycle progression in fibroblast cells. Cells were cultured with the indicated concentration of DP and complete medium (control group) for 24 h. The cell cycle was analyzed by propidium iodide staining. (a) The profiles of cell cycle analysis; (b) the percentage of cells in every phase and PI are shown (results are expressed as means ± SD of five determinations. ^*∗*^*P* < 0.05, ^*∗∗*^*P* < 0.01 compared with controls by Student's *t*-test).

Generally, fibroblast proliferation is closely related to the cell cycle. Therefore, we examined the cell cycle in DP-treated fibroblasts. The cell proliferation index (proliferation index, PI = S + G2/M) represents the number of proliferating cells in the cell population. The G2/M phase is the late stage of DNA synthesis, and DNA completes self-replication in S phase, which reflects the state of cell proliferation to some extent. Fibroblasts were treated with different concentrations (2, 3, and 4 *μ*g/mL) of DP for 24 h, and the cells treated with the complete medium were regarded as the control group. Using flow cytometry, we found that DP significantly reduced the number of cells in the G1 phase (*P* < 0.01) and increased the number of cells in the S (*P* < 0.01) and G2/M (*P* < 0.01) phases compared to that of the control group (Figure 3(a)). Additionally, PI was significantly increased (*P* < 0.01; Figure 3(b)). With DP treatment at 3 *μ*g/mL, the PI was the highest among all groups, suggesting that DP significantly accelerated the cell cycle.

### 3.3. DP Activated EGFR, ERK1/2, and PI3K Signaling Pathways

**Figure 4:**
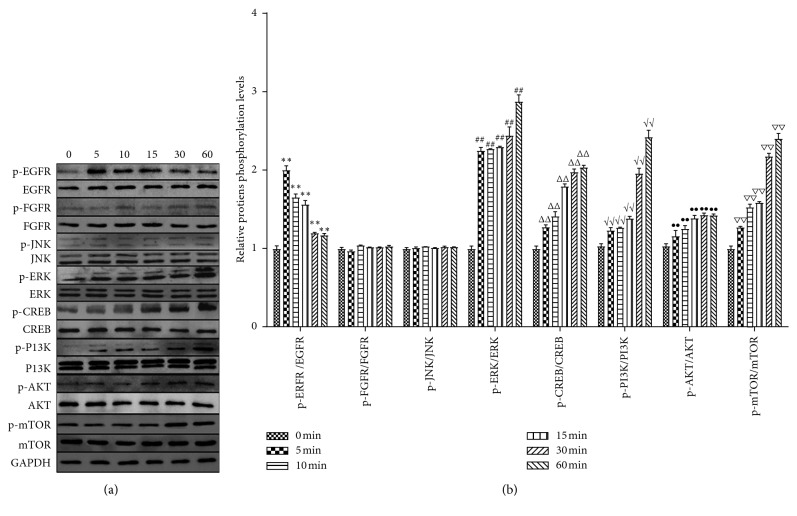
DP activated EGFR, ERK1/2, and PI3K signaling pathways in fibroblasts. (a) After the cells were treated with DP, the expression of p-EGFR/EGFR, p-FGFR/FGFR, p-ERK/ERK, p-JNK/JNK, p-CREB/CREB, p-PI3K/PI3K, p-Akt/Akt, and p-mTOR/mTOR was analyzed by immunoblotting. (b) Gray intensity was measured using ImageJ software at different time points, and the phosphorylation levels of related proteins were calculated and shown in Figure 4(b) (data are presented as means ± SD, *n* = 5 for each bar, single symbol, such as “^*∗*^,” indicates *P* < 0.05, and double symbols, such as “^*∗∗*^,” indicate *P* < 0.01 vs. control group).

To further investigate the molecular mechanisms responsible for the proliferative effects of DP in fibroblasts, p-EGFR/EGFR, p-FGFR/FGFR, p-ERK/ERK, p-JNK/JNK, p-CREB/CREB, p-PI3K/PI3K, p-Akt/Akt, and p-mTOR/mTOR were detected, respectively, after 3 *μ*g/mL DP treatment for 0, 5, 10, 15, 30, and 60 min. These proteins play major roles in regulating cell growth, survival, and differentiation. As shown in Figures 4(a) and 4(b), DP upregulated EGFR phosphorylation level in fibroblasts significantly at 5 to 60 min (*P* < 0.01); however, no change was found in p-FGFR (*P* > 0.05).

The EGFR signaling pathway downstream involves the MAPK and PI3K families. ERK1/2 and JNK, as MAPK family members, play important roles in the control of cell proliferation. In our further studies, p-ERK extremely increased after DP treatment in a time-dependent manner (*P* < 0.01). P-JNK had no obvious changes (*P* > 0.05). Because phosphorylated ERK1/2 is known to phosphorylate transcription factors, such as CREB, which regulates the transcription of genes involved in cell metabolism, growth, migration, and proliferation, we next examined the effects of DP on CREB phosphorylation. Similar to the effects that were previously observed, CREB phosphorylation was also significantly increased at 15, 30, and 60 min with DP (*P* < 0.01). On the other hand, we further assessed PI3K and its downstream protein (Akt/mTOR) phosphorylation levels. The level of p-PI3K showed a time-dependent increase after treatment with DP (*P* < 0.01). Similar results were also discovered in the levels of AKT/p-AKT and mTOR/p-mTOR (*P* < 0.01). These results demonstrated that DP activated EGFR, ERK1/2, and PI3K signaling pathways.

### 3.4. Related Inhibitors Suppressed DP-Induced Signaling Pathway Activation

**Figure 5:**
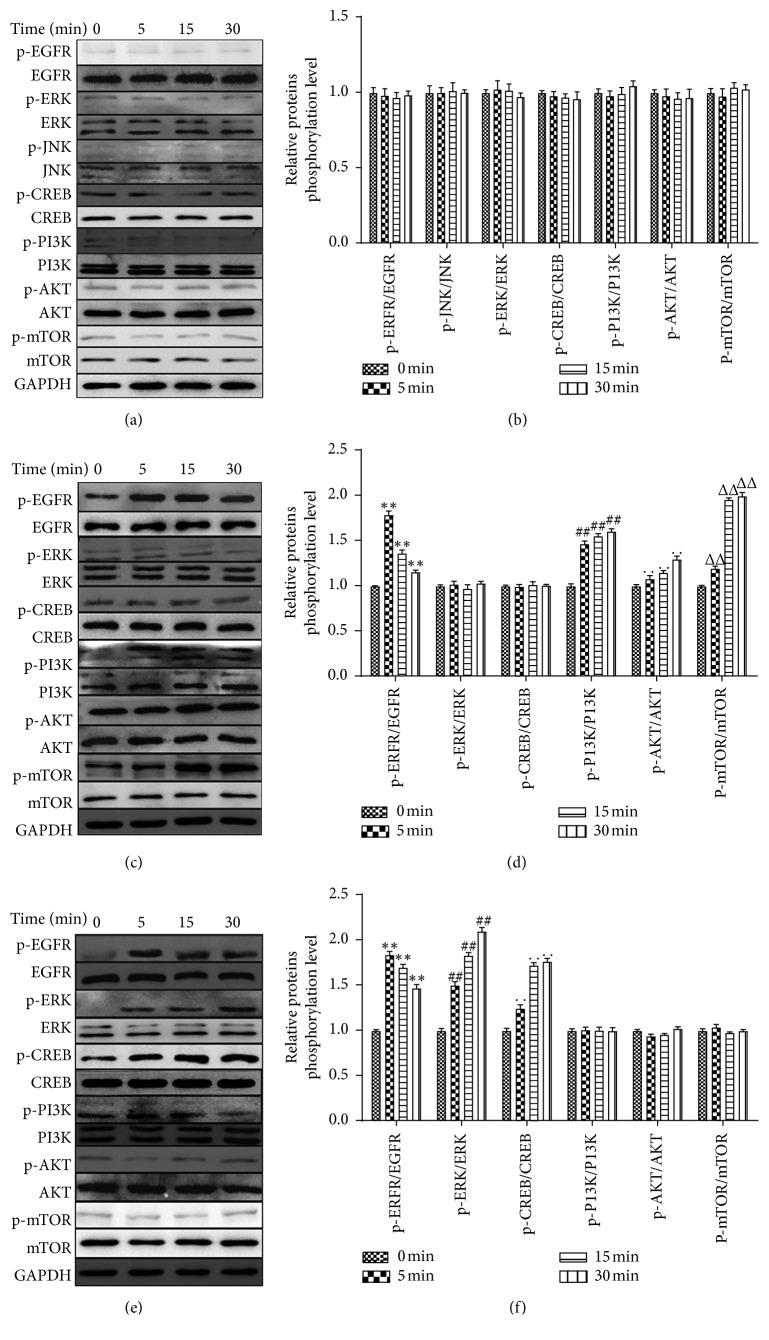
Related signaling pathway inhibitors suppressed DP-induced protein phosphorylation. (a, c, e) with AG1478, U0126, or LY294002 respective pretreatment for 12 h, cells were treated with 3 *μ*g/mL DP. Then, related protein expression was detected at 0, 5, 15, and 30 min after DP treatment. (b, d, f) Phosphorylation levels of related proteins were determined (data are presented as means ± SD, *n* = 5 for each bar. Single symbol, such as “^*∗*^,” indicates *P* < 0.05, and double symbols, such as “^*∗∗*^,” indicate *P* < 0.01 vs. control group).

According to the above experimental results, we added signaling pathway inhibitors into cell-cultured plates to pretreat fibroblasts. The aim of this was to determine the involvement of EGFR, ERK1/2-CREB, and PI3K/Akt/mTOR activation in DP-induced protein phosphorylation. The results showed that EGFR inhibitor (AG1478) inhibited DP-promoted EGFR phosphorylation, and then the ERK and PI3K families were not activated by DP as they were in the control group (*P* > 0.05, Figures 5(a) and 5(b)). The phosphorylation levels of JNK did not increase compared to those of the control group (*P* > 0.05, Figures 5(a) and 5(b)). In Figures 5(c) and 5(d), the cells pretreated by ERK inhibitors (U0126) significantly depressed DP-induced ERK and CREB phosphorylation, which had no changes compared to that of the control group (*P* > 0.05). However, DP-induced EGFR and PI3K phosphorylation was still observed (*P* < 0.01). As shown in Figures 5(e) and 5(f), with the pretreatment of PI3K signaling pathway inhibitor LY294002, DP no longer activated PI3K and the downstream Akt/mTOR phosphorylation (*P* > 0.05 vs. control group). Similarly, EGFR, ERK1/2, and CREB were activated markedly compared with the control group (*P* < 0.01). In conclusion, we believe that EGFR resides in the upstream of ERK1/2-CREB and PI3K/Akt/mTOR to function as the receptor of DP to regulate cellular signaling.

### 3.5. Inhibitors Blocked DP-Induced Fibroblast Cell Proliferation

**Figure 6:**
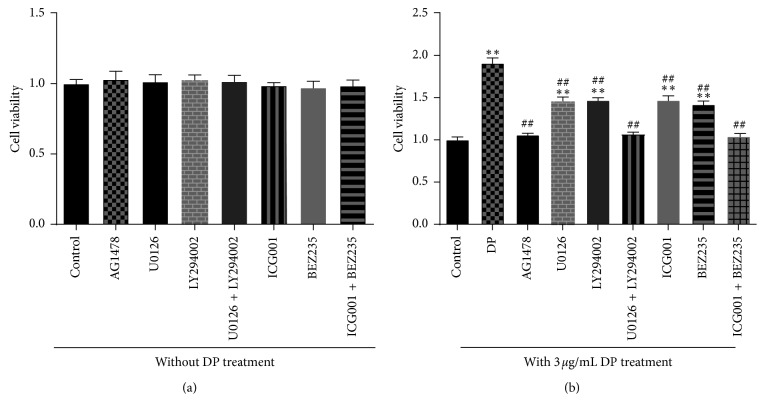
Inhibitors suppressed DP-induced fibroblast cell proliferation. (a) The effects of inhibitors on fibroblast proliferation were detected using CCK-8. (b) In subsequent experiments, the experimental groups were divided into DP-treated group without inhibitor pretreatment (DP group), DP + AG1478, DP + U0126, DP + LY294002, DP + U0126 + LY294002, DP + ICG001, DP + BEZ235, and DP + ICG001 + BEZ235. The cells without DP treatment were used as the control group. Cells were pretreated with inhibitors individually for 12 h and then were cultured in a complete medium containing 3 *μ*g/mL DP for 24 h. Finally, the cell viability was assessed using CCK-8. The results showed that DP-stimulated cell proliferation was inhibited by related inhibitors (data are presented as means ± SD, *n* = 15 for each bar, ^*∗∗*^*p* < 0.01 vs. control group, ^##^*p* < 0.01 vs. DP-treated group without inhibitor pretreatment).

To investigate the involvement of EGFR, ERK1/2-CREB, and PI3K/Akt/mTOR activation in cellular proliferation by DP, we measured the DP-induced fibroblast proliferation using related inhibitors. In our preexperiments, we found that inhibitors did not affect cell proliferation when cells were not treated by DP (*P* > 0.05, Figure 6(a)). The results of subsequent detections are shown in Figure 6(b), which demonstrates significantly low cell viability due to the inhibition of EGFR, ERK1/2, PI3K-AKT, CREB, and mTOR (92%, 49%, 47%, 48%, and 50%, respectively) compared to the DP-treated group without inhibitor pretreatment. DP-induced cell proliferation was almost completely inhibited with AG1478 (*P* < 0.01 vs. DP group, *P* > 0.05 vs. control group). However, when U0126 and LY294002 were used alone, the cell activity was still significantly increased compared to that of the control group (*P* < 0.01). This result suggested that DP-induced cell proliferation may be closely associated with the ERK and PI3K pathways simultaneously. Therefore, in further work, we determined that the cell proliferation was inhibited completely using U0126 and LY294002 together (*P* < 0.01 vs. DP group, *P* > 0.05 vs. control group), and the cell viability was similar to that in the AG1478-treated group (*P* > 0.05). The same results were also found in cells pretreated by ICG001 and BEZ235 together, which almost abolished DP-induced proliferation (*P* < 0.01 vs. DP group, *P* > 0.05 vs. control group).

Taken together, our data demonstrated that inhibitors of EGFR, ERK1/2, PI3K/Akt, CREB, and mTOR suppressed DP-induced fibroblast proliferation, thereby suggesting that DP stimulated fibroblast proliferation via the EGFR/ERK/CREB and EGFR/PI3K/Akt/mTOR signaling pathway.

## 4. Discussion

In association with reepithelialization, the restoration of the dermis occurs by the migration and proliferation of fibroblasts. The response of fibroblasts during wound healing determines the outcome of tissue repair. In response to wounding, macrophages and fibroblasts release growth factors that lead to further fibroblast migration and proliferation [29]. Our experimental results showed that DP was effective in promoting the proliferation of fibroblasts. We also found that when DP was more than 3 *μ*g/mL, DP inhibited fibroblast proliferation to some extent compared with 3 *μ*g/mL DP-treating cells. Many studies have demonstrated that the extracts of natural plants accelerate cell proliferation, playing a positive role when used within an optimal concentration range. However, the negative effects of using the same extracts are often observed when their concentrations are too high [30]. We hypothesize that this may be related to cell tolerance to drugs. The present study determined the effects of DP on the cell cycle and the mechanism of DP-induced fibroblast proliferation for the first time. It is well known that the cell cycle and proliferation are closely linked. The G1 phase refers to the period from mitosis to DNA replication, also known as the presynthesis phase. The S phase is called the DNA synthesis phase, which involves the synthesis of DNA as well as the production of related proteins. The G2 phase is the late stage of DNA synthesis, and the M phase is mitosis, which reflects the state of cell proliferation to some extent [31]. In many studies, the transition of the cells from the G1 to the S and G2/M phases is crucial for cell proliferation, and they all demonstrated that one possible mechanism is that the activation of static cells is transited from the G1 phase to the S phase, accelerating DNA synthesis and thereby promoting cell proliferation [32, 33]. In our study, we found that the PI (PI = S + G2/M) was significantly increased after DP treatment.

To further investigate the mechanism of DP-induced fibroblast proliferation, related proteins were detected by western blot. EGFR and FGFR are widely distributed in fibroblast membranes, and their activation leads to the activation of a series of pathways. Many studies have reported that EGFR and FGFR activation play important roles in cell processes such as cell growth, proliferation, and differentiation [34, 35]. In the detections of EGFR and FGFR after DP treatment, we found that EGFR phosphorylation level was significantly increased, but FGFR has no obvious change. Thus, we speculated that EGFR may be the molecular target of DP acting on fibroblasts. As described above for the DP structure, which is similar to flavonoids, it has reported that flavonoids stimulated EGFR activation [23]. In the reports of Li and Waseem, they demonstrated that the activation of EGFR primarily triggered the MAPK pathway, the PI3K pathway, and some other cell proliferation pathways [36, 37]. The MAPK family mainly includes ERK1/2, p38, JNK, and ERK5, and ERK1/2 and JNK, in particular, play important roles in the control of cell proliferation [38]. With DP treatment, we found that only ERK phosphorylation levels increased significantly. In quiescent cells, all components of the ERK1/2 module have a cytoplasmic localization, but upon extracellular stimulation, a significant proportion of ERK1/2 accumulates in the nucleus [39]. CREB, a transcription factor, is activated with phosphorylated ERK transmitted into the nucleus. This activates genes associated with cell proliferation [26, 40]. In the present study, the detection of the CREB phosphorylation level demonstrated that DP upregulated p-CREB in fibroblasts. In addition, we found that the PI3K signaling pathway and downstream Akt/mTOR were also activated by DP treatment. This pathway is involved in cell proliferation, differentiation, and apoptosis. The PI3K activation results in PIP3 (second messenger) production on the plasma membrane, and PIP3 combines with the signaling protein AKT containing the PH domain in the cell and activates the Akt signaling pathway. Phosphorylated Akt can prevent the negative regulation of the small G protein Ras homolog enriched in the brain (Rheb), thereby activating Rheb-enriched mTOR. PI3K/AKT/mTOR signaling contributes to various processes that are crucial in mediating many aspects of cellular function, involving nutrient uptake, anabolic reactions, cell growth, and proliferation [41, 42]. Their conclusions are consistent with ours.

**Figure 7:**
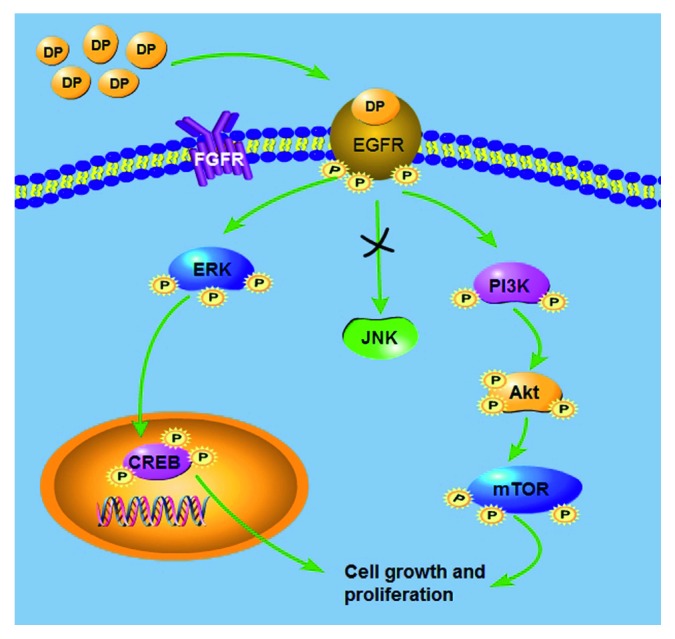
In the DP-treated cells, DP preferentially recognized and activated EGFR receptors on the cell surface. Phosphorylated EGFR stimulated downstream ERK and PI3K signaling pathway activation, which triggered their downstream protein activation. Finally, the cell proliferation was accelerated.

Moreover, we investigated the relationship between cell proliferation and phosphorylated proteins, such as the EGFR, ERK/CREB, and PI3K/Akt/mTOR signaling pathways. We proved that EGFR is highly involved in the activation of ERK1/2, PI3K, Akt, CREB, and mTOR factors with DP stimulus by showing that AG1478 inhibits ERK1/2, PI3K, Akt, CREB, and mTOR activation. Moreover, we demonstrated that DP-induced fibroblast proliferation was abolished with the inhibition of EGFR. These results verified our previous speculation, which is that DP preferentially recognizes EGFR on the surface of fibroblasts. Recent reports have highlighted that celecoxib induced fibroblast proliferation through the EGFR signaling pathway [43]. Furthermore, U0126 suppressed the ERK/CREB phosphorylation level and LY294002 inhibited PI3K/Akt/mTOR phosphorylation level, respectively, but neither of them affected DP-induced EGFR activation. We also found that U0126 or LY294002 partially reduced DP-induced cell proliferation. We thus determined the mechanism by which DP affects cell proliferation via EGFR downstream ERK and PI3K signaling pathways together. Previous studies have reported that EGFR activation stimulates the phosphorylation of ERK and PI3K [37, 44]. Finally, we examined DP-induced fibroblast proliferation using ICG001 and BEZ235 together. The results showed that they completely suppressed DP-induced cell proliferation. Taken together, our findings suggest that DP-promoted fibroblast proliferation is stimulated by p-EGFR-induced activation of the ERK1/2-CREB and PI3K/Akt/mTOR pathways (Figure 7). Recent study has reported that some kinds of common cytokines, such as EGF, PDGF-AA, VEGF, and bFGF, significantly stimulate dermal fibroblast proliferation [45]. Moreover, EGF gel is a well-known wound treatment in clinical settings. Another study showed that EGF stimulated the activation of EGF receptors and the selective activation of major signaling pathways during mitosis. In other words, activation of EGFR plays a key role in cell proliferation [46]. This is consistent with our findings. In addition, the above cytokines are beneficial for fibroblasts to produce type I collagen, which is the main component in skin fibrosis, wound healing, tissue remodeling, and skin aging [47]. Accelerated proliferation of fibroblasts is the most important part of wound healing, and we have demonstrated that DP significantly promotes fibroblast proliferation. Thus, we speculate that DP may stimulate the synthesis and remodeling of the extracellular matrix, and we will further detect the expression of related matrixes produced by fibroblast with the treatment of DP.

With the rapid development of medical field, there is increasing research on the application of plants in medicine, and to date, many natural plants have been applied in the clinical treatment of diseases. Currently, studies have reported that natural plant extracts could treat neurological disease [48], tumors [49], and dermatitis [50]. Some natural extracts have been reported to have an edge over synthetic drugs, for example, showing fewer side effects and minimizing drug resistance [51]. The skin is a sensitive and protective organ across the entire human body; therefore, our study on wound treatment is meaningful. Although the role of dragon's blood in wound healing has been previously demonstrated [52], our study is the first to describe the effects of DP on the cell cycle and the mechanism of promoting fibroblast proliferation using in vitro assays. Hence, this study provides strong evidence for further research in clinical cases whose wounds are difficult to heal in the future. In conclusion, our present work provided a new basis for wound healing.

## 5. Conclusion

Taken together, our data demonstrated that inhibitors of EGFR, ERK1/2, PI3K/Akt, CREB, and mTOR suppressed DP-induced fibroblast proliferation, thereby suggesting that DP stimulated fibroblast proliferation via the EGFR/ERK/CREB and EGFR/PI3K/Akt/mTOR signaling pathway.

## Data Availability

The data used to support the findings of this study are included within the article.
