# Corrigendum to “Dracohodin Perochlorate Stimulates Fibroblast Proliferation via EGFR Activation and Downstream ERK/CREB and PI3K/Akt/mTOR Pathways In Vitro”

**DOI:** 10.1155/2020/4531097

**Published:** 2020-05-28

**Authors:** Lin Liu, Xiaowen Jiang, Wenhui Yu

**Affiliations:** ^1^College of Veterinary Medicine, Northeast Agricultural University, Harbin 150030, China; ^2^Key Laboratory of the Provincial Education Department of Heilongjiang for Common Animal Disease Prevention and Treatment, Harbin, China

In the article titled “Dracohodin Perochlorate Stimulates Fibroblast Proliferation via EGFR Activation and Downstream ERK/CREB and PI3K/Akt/mTOR Pathways In Vitro” [1], there was an error in the Acknowledgments section. The corrected section appears below.
